# Axonal regeneration and hyperglycemia in wound healing: a review on the role of activin A, TNFRSF10B, and Synaptophysin

**DOI:** 10.1007/s11033-026-11941-5

**Published:** 2026-05-13

**Authors:** Jaylan Patel, Vikrant Rai

**Affiliations:** Department of Translational Research, College of Osteopathic Medicine of the Pacific, Western University of Health Sciences, 309 E. Second Street, Pomona, CA 91766 USA

**Keywords:** Axonal regeneration, Wound healing, Nonhealing diabetic foot ulcers, Activin A, TNFRSF10B, Synaptophysin

## Abstract

Diabetic foot ulcers are a major cause of morbidity in patients with diabetes mellitus and remain challenging to treat despite advances in wound care. Growing evidence suggests that impaired axonal regeneration, along with chronic inflammation, impaired angiogenesis, and altered extracellular remodeling, is an important mechanism contributing to the chronic, nonhealing nature of cutaneous wounds. However, the mediators involved and the mechanisms by which they impair wound healing in the presence of hyperglycemia are poorly understood. Peripheral nerves coordinate inflammation, angiogenesis, and extracellular matrix remodeling during repair, and their dysfunction in diabetes disrupts these processes. This review examines how hyperglycemia and oxidative stress impair neuroregeneration ( mainly axonal and nerve regeneration), highlighting the downstream consequences for vascular and structural repair. Particular attention is given to the altered expression of activin A (associated with nerve growth and neural inflammation), TNFRSF10B (associated with neuronal damage and apoptosis, and synaptophysin (a marker of synaptic vesicles and involved in nerve regeneration, particularly in the early stages of axonal regrowth) as representative mediators linking neuronal injury to defective wound healing. By integrating findings across neural, vascular, and inflammatory pathways, this review supports impaired neuronal regeneration as an important contributor to nonhealing diabetic foot ulcer pathogenesis to identify potential molecular targets that may improve healing outcomes.

## Introduction

Diabetes mellitus is a chronic disease characterized by elevated glucose levels, or hyperglycemia, and remains one of the most common disorders and leading causes of death in the United States [1]. It is associated with multiple complications that significantly reduce quality of life. Among these, diabetic foot ulcers (DFUs) are particularly prevalent, arising from a combination of peripheral neuropathy, repetitive trauma, and comorbidities common in diabetic patients [2]. Despite advances in wound care consisting of offloading pressure, antibiotics, debridement, wound care, and surgical intervention to improve blood flow [3], DFUs frequently recur and often fail to heal, resulting in amputation. This emphasizes the need to better understand the mechanisms underlying delayed or impaired healing. An often-underrecognized factor in the development and recurrence of DFUs is diabetic peripheral neuropathy.

The peripheral nervous system plays critical roles in sensation, motor function, intercellular communication, and regeneration [4]. During wound healing, peripheral nerves contribute to repair by releasing neuropeptides such as Substance P (SP), calcitonin gene-related peptide (CGRP), neuropeptide Y (NPY), and vasoactive intestinal peptide (VIP). Neurotransmitters such as norepinephrine (NE) and acetylcholine (ACh), and growth factors including nerve growth factor (NGF), brain-derived neurotrophic factor (BDNF), and fibroblast growth factor-2 (FGF-2) regulate inflammation, angiogenesis, and extracellular matrix (ECM) remodeling during wound healing [5, 6]. When this neural input is lost, coordination of these processes is disrupted, predisposing tissue to recurrent trauma and delayed healing. In diabetes, hyperglycemia and oxidative stress further alter the expression of key neuroregenerative mediators that are essential for effective wound healing [7]. Our recent findings of decreased levels of TNFRSF10B, activin A, and synaptophysin with hyperglycemia in skin samples of diabetic wounds in Sprague Dawley (SD) rats suggested a role of these factors in delayed wound healing in diabetes. It is important to note that in acute conditions, hyperglycemia increases the expression of TNFRSF10B and decreases in chronic conditions, as evidenced in our study using in-vitro studies using fibroblasts [8]. Activin A, synaptophysin, and TNFRSF10B are particularly important regulators of neurovascular repair. These three mediators were selected to represent complementary components of the impaired wound-healing environment under hyperglycemic conditions. Activin A reflects pathways involved in tissue repair, angiogenesis, and ECM remodeling, while TNFRSF10B represents apoptosis and inflammatory signaling associated with cellular injury [8].

Activin A is a protein from the TGF-β superfamily, produced by multiple cell types, including macrophages, fibroblasts, keratinocytes, and endothelial cells. Activin A acts as a hormone, growth factor, and cytokine and is involved in physiological processes like cell differentiation, proliferation, and wound healing [9]. Activin A has been associated with inflammation, angiogenesis, and ECM remodeling, which are essential for creating a permissive environment for axonal growth [10]. Synaptophysin plays a role in neurotransmitter release and regulates synaptic vesicle endocytosis [11]. Activin A has been included because it acts as an injury-related signal that promotes Schwann cell activity, modulates inflammation, and acts synergistically with NGF to regulate neuropeptide expression, while NGF is a neurotrophin that primarily promotes neuronal survival and axonal outgrowth [12]. BNDF is primarily a promoter of neurite outgrowth and axonal regeneration but is heavily involved in activity-dependent regeneration [13].

Synaptophysin, a membrane glycoprotein found in the presynaptic vesicles of neurons and endocrine cells, reflects neuronal connectivity and regenerative activity, serving as an indicator for synapse formation [14]. It plays a crucial role in synaptic vesicle recycling and endocytosis by managing the trafficking of synaptobrevin-2 (VAMP2). In contrast to established neurotrophic mediators such as NGF and BDNF, synaptophysin is included as a marker of neuronal density and synaptic integrity. It provides a direct measure of neuroregenerative status rather than acting as a primary signaling mediator [15]. In other words, NGF promotes the survival and outgrowth of neurons, while synaptophysin is a marker for synaptic vesicle formation and functional reconnection. Further, BNDF promotes nerve regeneration, neuronal survival, and synaptic plasticity by inducing synaptophysin-dependent synapse formation [16]. Thus, evaluating synaptophysin directly evaluates BNDF activity. This distinction allows assessment of neuronal presence and regenerative activity within the wound environment. Although its role in diabetic wound healing remains less well characterized, incorporating synaptophysin enables integration of structural neuronal changes with molecular signaling pathways and highlights an area that warrants further investigation.

TNFRSF10B, also known as death receptor 5 (DR5), is a protein expressed on a variety of cell types, including epithelial cells, endothelial cells, immune cells, and fibroblasts. TNFRSF10B acts as a receptor for the TRAIL ligand, and once activated, it is involved in triggering programmed cell death (apoptosis). It is a member of the TNF-receptor superfamily and plays a role in immune responses, tumor suppression, and diseases like Parkinson’s [17]. TNFRSF10B regulates apoptosis and inflammatory resolution, which can influence neuronal survival under stress conditions [18], the reason to include TNFRSF10 in this review, though not directly involved in nerve regeneration. Further, the role of TNFRSF10 in nerve regeneration in association with immune response, inflammation, and apoptosis has not been discussed in the recent literature. So it is important to highlight the role of TNFRSF10B, which is significantly increased in diabetes and plays a role in diabetic complications, in wound healing in diabetic wounds.

Another reason to include these mediators in this review is the decreased expression of these mediators in diabetic wounds in SD rats, which were not subjected to exercise [8]. These mediators are therefore derived from both neuronal and non-neuronal cell populations within the wound microenvironment. Together, they provide a framework for understanding how structural, microenvironmental, and cell survival mechanisms converge to regulate axonal regeneration in diabetic wound healing. Dysregulation of these mediators under diabetic conditions contributes to impaired tissue repair and chronic ulcer formation. The role of activin A, TNFRSF10B, and synaptophysin has been discussed in various aspects, but their role in wound healing in diabetics, especially DFUs, has not been completely understood. Together, these factors provide a framework to examine how hyperglycemia disrupts interconnected processes of neuronal regeneration, cell survival, and tissue repair.

This narrative review summarizes the role of axonal regeneration in normal wound repair, describing how diabetes disrupts the expression of key molecular mediators and connects these alterations to impaired inflammation, angiogenesis, and ECM remodeling. Axonal regeneration will be referred to as the regrowth of peripheral nerve fibers into the wound bed, a key component of neurocutaneous repair. This review focuses on peripheral axonal regeneration within the wound bed as a component of neurocutaneous repair, rather than broader diabetic neuropathy. Outlining the mechanisms involved in nonhealing of DFUs and the roles of these mediators will allow us to highlight impaired neuronal regeneration as a key contributing factor in DFU formation and nonhealing patterns. By establishing this concept, we can highlight potential therapeutic targets to improve patient outcomes.

## Physiological wound healing

**Fig. 1:**
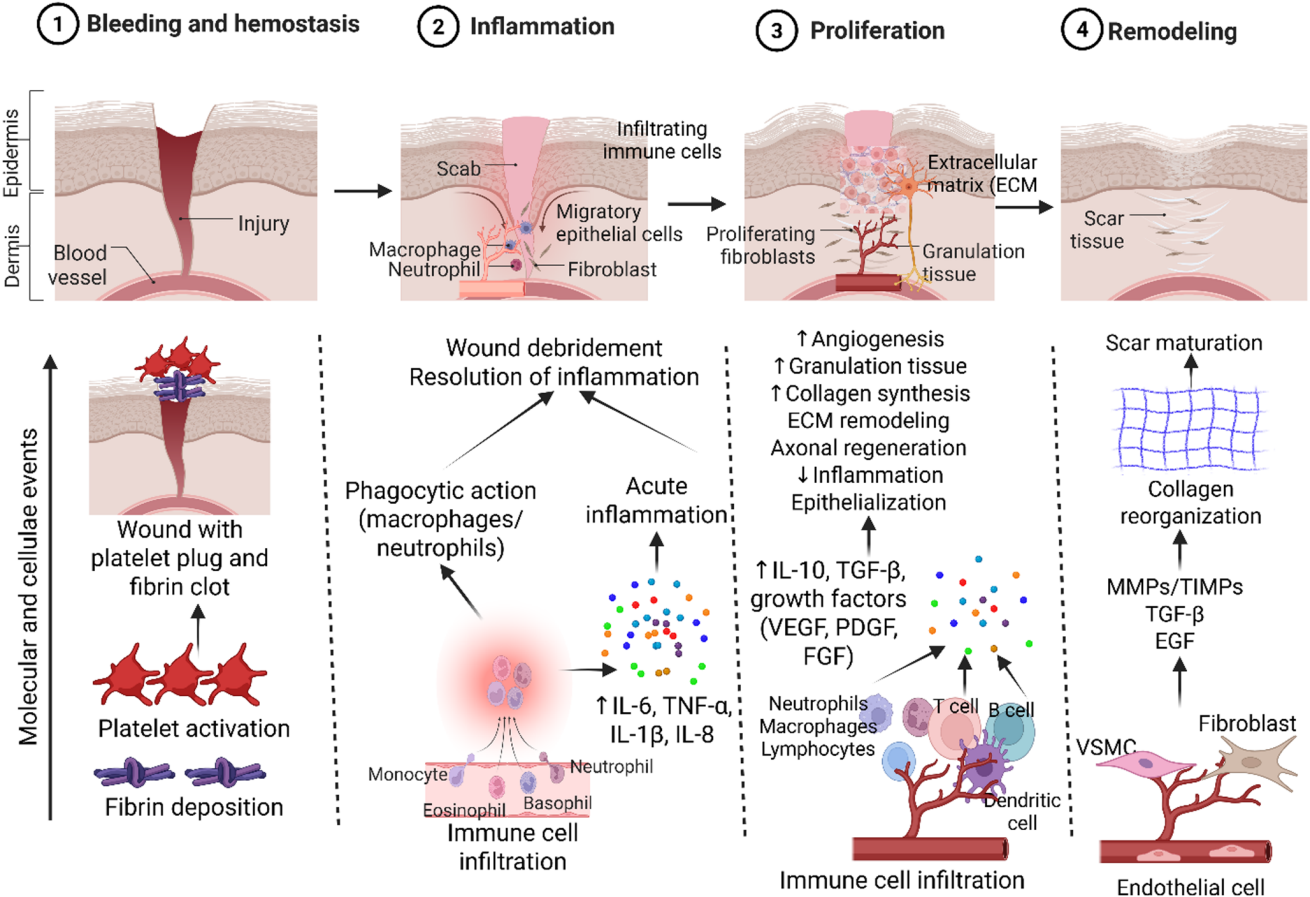
Cellular and molecular events during the four phases of wound healing. Hemostasis (stopping bleeding with clots), Inflammation (cleaning debris/bacteria with white cells), Proliferation (rebuilding with new tissue/granulation), and Remodeling/Maturation (strengthening the new tissue and scar) work together to restore skin integrity, starting immediately after injury and continuing for months or years. Interleukin (IL), tumor necrosis factor alpha (TNF-α), transforming growth factor beta (TGF-β), platelet-derived growth factor (PDGF), vascular endothelial growth factor (VEGF), fibroblast growth factor (FGF), epidermal growth factor (EGF). Created in BioRender. Rai, V. (2026) https://BioRender.com/8g8wbna

Physiological wound healing is a complex process that involves hemostasis, inflammation, proliferation, and remodeling. Immediately after injury, hemostasis stops bleeding through platelet aggregation and clot formation, providing a temporary ECM scaffold [19]. Next is the inflammatory phase, characterized by the infiltration of neutrophils and macrophages that clear debris, secrete cytokines, and initiate the repair cascade [20]. The proliferative phase is defined by angiogenesis, fibroblast proliferation, and keratinocyte migration. New vasculature restores oxygen delivery, while fibroblasts deposit ECM components, such as collagen and fibronectin, to establish granulation tissue. Finally, during the remodeling phase, ECM undergoes maturation and reorganization, restoring tensile strength and tissue integrity [19, 21] (Fig. 1). Effective wound repair requires tightly regulated inflammation, angiogenesis, and ECM remodeling. In contrast, persistent inflammation, impaired angiogenesis, and defective ECM remodeling can disrupt this coordination. This can result in a chronic, nonhealing wound marked by hypoxia, fibrosis, and loss of structure.

Nerve injury during wounds occurs via deep cuts, lacerations, punctures, stretching, or compression, causing numbness and weakness of muscles. Nerve regeneration, which is needed for wound repair, primarily occurs during the proliferative phase of wound healing. While initial injury responses start earlier, functional repair, including Schwann cell activation, axon sprouting, and formation of bands of Bungner for guidance, peaks as the tissue rebuilds alongside angiogenesis and fibroplasia [22].

## Neuronal regeneration and wound healing

Axonal regeneration is a critical yet often underappreciated component of the wound healing process. Sensory and autonomic nerves contribute to tissue repair through the secretion of neuropeptides, neurotransmitters, and growth factors (Table 1) that influence key healing mechanisms, including inflammation, angiogenesis, and cellular proliferation [23].

**Table 1:** Role of neuropeptides, neurotransmitters, and growth factors in wound healing

Mediator	Class	Role in wound healing	Wound-healing phase
Substance P [5, 24]	Neuropeptide	Promotes angiogenesis and inflammation	Inflammation, Proliferation
Calcitonin Gene-Related Peptide[25]	Neuropeptide	Promotes resolution of inflammation and tissue repair.	Inflammation, Proliferation
Neuropeptide Y [26]	Neuropeptide	Modulates inflammation, promotes angiogenesis, and strengthens the ECM.	Inflammation, Proliferation, Remodeling
Vasoactive Intestinal Peptide [27]	Neuropeptide	Anti-inflammatory and promotes cell proliferation.	Inflammation, Proliferation
Norepinephrine [28]	Neurotransmitter	Regulates vascular tone and immune activation.	Inflammation, Proliferation
Acetylcholine [29]	Neurotransmitter	Promotes keratinocyte migration and angiogenesis.	Proliferation
Nerve Growth Factor [30]	Growth Factor	Promotes neuron and vascular regeneration.	Proliferation, Remodeling
Brain-Derived Neurotrophic Factor [31]	Growth Factor	Promotes neuron regeneration.	Proliferation, Remodeling
Fibroblast Growth Factor-2 [32]	Growth Factor	Promotes cell proliferation, angiogenesis, and tissue remodeling.	Proliferation, Remodeling

Neuropeptides, neurotransmitters, and growth factors are involved in various phases of healing, including inflammation, cell proliferation, and tissue remodeling. These factors influence immune cells, repair cells, and the growth factor network working together to create a supportive environment for healing, promoting the repair of damaged tissues and the restoration of normal function

Inflammation, angiogenesis, ECM remodeling, and nerve regeneration collectively regulate the regeneration of damaged tissue, and re-epithelialization emphasizes the importance of neural signaling in effective wound repair. An indicator of neuronal involvement in healing is reinnervation, the regrowth of nerve fibers into the wound bed. Studies have demonstrated that nerve fiber density positively correlates with re-epithelialization, indicating a relationship between axonal regeneration and wound closure [33]. While the precise mechanisms are not yet completely understood, this association highlights the importance of a functional peripheral nervous system during healing. Tissue regeneration after injury ultimately depends on a coordinated interplay of multiple biological processes, among which axonal regeneration, ECM remodeling, and angiogenesis serve as three central mechanisms driving effective repair [34].

Inflammation plays a critical role in wound healing. Acute inflammation mediated by proinflammatory cytokines, including interleukin (IL)-6, IL-8, IL-1β, and tumor necrosis factor (TNF)-α, clears the debris and prepares the wound for healing. Immune cells, including macrophages, neutrophils, dendritic cells, and lymphocytes, also contribute to wound healing (Fig. 1). However, chronic inflammation is detrimental to the wound and leads to a nonhealing chronic wound [20, 35]. Furthermore, impaired axon regeneration can lead to chronic inflammation during wound healing because the absence of proper nerve signaling disrupts the normal, self-resolving inflammatory process. Nerve damage can prevent the timely and effective resolution of inflammation, leading to a persistent, damaging inflammatory state that hinders tissue repair [36].

Angiogenesis, the growth of new blood vessels, is critical for restoring perfusion and meeting the metabolic demands of regenerating tissue. After an acute injury, both nerves and vessels are damaged, resulting in pain and localized hypoxia. Angiogenesis counteracts this by re-establishing oxygen and nutrient delivery to the wound bed [37]. Adequate vascularization supports both cutaneous and neuronal healing, as axonal survival and outgrowth are highly dependent on sufficient oxygenation and trophic support. Conversely, when angiogenesis is disrupted, nerve repair and overall tissue healing become irregular, leading to delayed or incomplete restoration of tissue integrity. This is because nerves and blood vessels often run in parallel (neurovascular congruency) and share similar guidance cues and growth factors, including vascular endothelial growth factor (VEGF), nerve growth factor (NGF), and substance P, during development and repair [38].

ECM remodeling is another regenerative process that provides the structural framework necessary for tissue repair and guides cellular behavior. ECM not only supports cell migration, adhesion, and differentiation but also serves as a reservoir for growth factors and signaling molecules that regulate the healing microenvironment. Granulation tissues and ECM also provide a stable bed for angiogenesis. Remodeling is one of the final phases of repair, restoring tissue integrity through collagen deposition, crosslinking, and structural reinforcement [39]. Proper ECM remodeling is therefore essential for stable and functional tissue regeneration. Peripheral nerves contribute to ECM remodeling during cutaneous healing by releasing growth factors, neuropeptides, and neurotransmitters that regulate the activity of fibroblasts, myofibroblasts, and other cells responsible for ECM synthesis and degradation. Impaired axonal regeneration may disrupt this process by altering the availability of these mediators, which have been shown to influence fibroblast proliferation, migration, and collagen production in experimental wound models [40]. Sensory nerves (expressing Substance P and CGRP) and autonomic nerves (expressing Neuropeptide Y) interact with fibroblasts to promote proliferation, migration, and differentiation into myofibroblasts. These myofibroblasts are responsible for ECM contraction and deposition of collagen I/III, crucial for wound closure. Substance P enhances the function of MMPs, such as MMP-2, within fibroblasts, accelerating ECM degradation and remodeling [40]. In addition, neural signaling has been associated with the regulation of matrix metalloproteinases (MMPs), key enzymes involved in ECM turnover. Consequently, impaired axonal regeneration may contribute to chronic wounds or excessive scarring [41].

Finally, axonal regeneration is an important component of peripheral nerve repair and functional recovery. Schwann cells, specialized glial cells of the peripheral nervous system, are key drivers of this process. Following nerve injury, Schwann cells undergo a phenotypic transformation into repair cells that proliferate, secrete inflammatory mediators and neurotrophic factors, and help guide regenerating axons toward their targets [42]. These cells also participate in wound healing through paracrine signaling, linking neural and cutaneous repair mechanisms. Reduced Schwann cell density has been associated with delayed wound contraction and impaired epithelial proliferation, highlighting their essential role in the regenerative cascade [43]. Given this connection, Schwann cell dysfunction has been implicated in impaired wound healing in peripheral neuropathies, and targeting Schwann cells has emerged as a promising therapeutic strategy to restore regenerative capacity in conditions such as DFUs [44].

## Mediators of nerve regeneration and wound healing

**Fig. 2:**
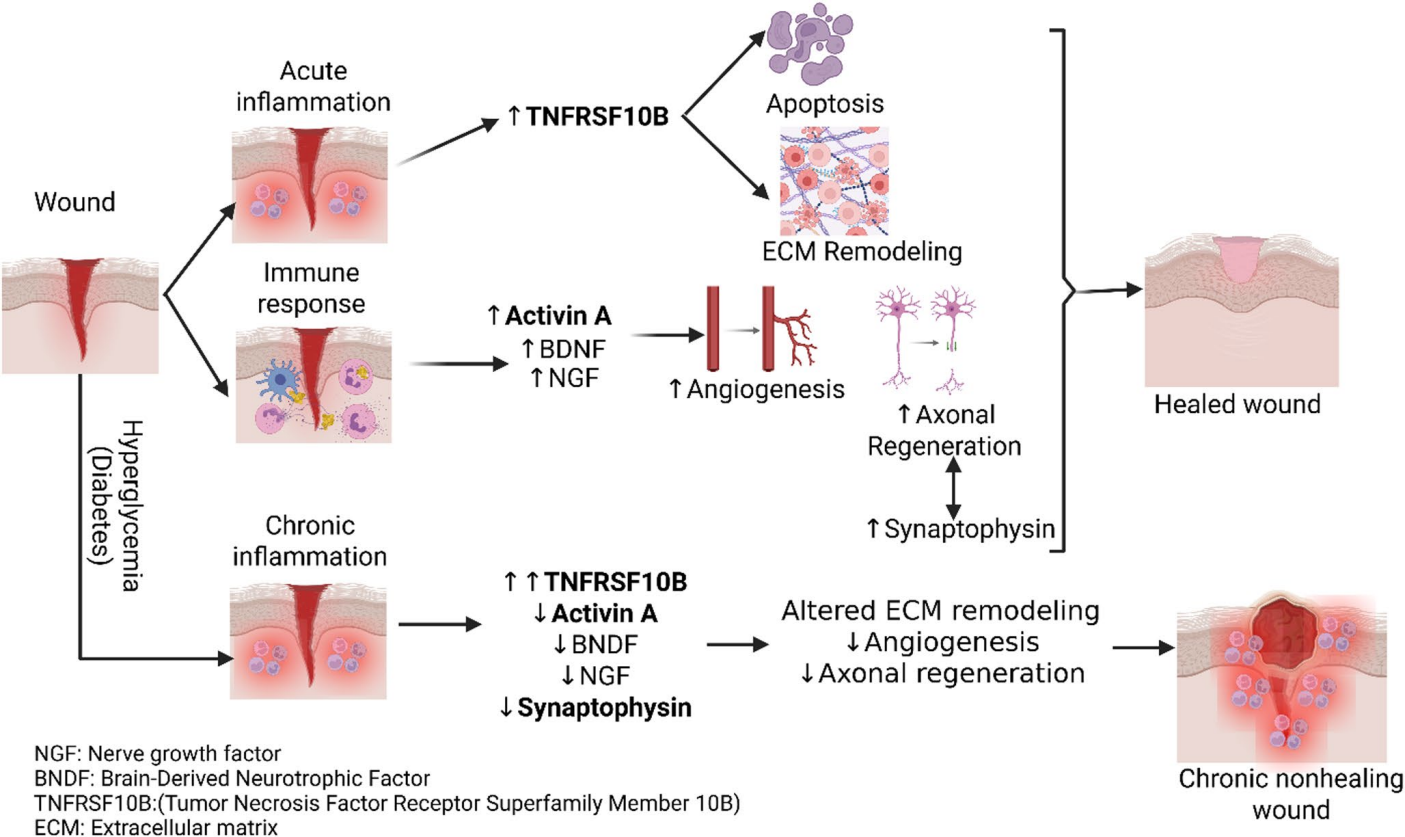
Mediators of axonal regeneration and wound healing. Created in BioRender. Lal, M. (2026) https://BioRender.com/8vumtgg

Several molecular mediators play critical roles in neuronal regeneration during normal wound healing. Activin A is a promoter of axonal regeneration and a multifunctional growth factor that accelerates cutaneous healing and supports neuronal recovery following injury [45]. Activin A plays a complex role in wound healing and nerve regeneration, promoting skin repair but also contributing to scarring (Fig. 2). In nerve regeneration, it enhances functional recovery after injuries, such as spinal cord damage, by inhibiting cell death and promoting the reconstruction of neural circuits. In wound healing, it increases granulation tissue, collagen deposition, and re-epithelialization, which can lead to faster healing but also more significant scarring [46]. It is stimulated by inflammatory signals, leading to fibrosis and tissue remodeling [47]. Elevated levels of activin A are commonly observed in inflammatory and ischemic conditions. Activin A is rapidly upregulated in fibroblasts, keratinocytes, and macrophages following an acute injury. Elevated levels of Activin A are effective in promoting wound healing in acute conditions, but its sustained chronic elevation in chronic inflammation settings is associated with excessive scar formation (due to excessive proliferation of fibroblasts and highly cross-linked collagen deposition), impaired healing, impaired vascular remodeling, and delayed tissue recovery. This underscores its role as both mediators of normal healing and contributors to pathological remodeling when dysregulated [10, 48].

Synaptophysin serves as a useful marker of synapse density and neuronal activity [14]. While synaptophysin has a defined role in synaptic vesicle dynamics, it is primarily used as a marker of neuronal density and synaptic integrity. This reflects regenerative activity rather than directly mediating wound healing processes. During nerve regeneration, especially in the peripheral nervous system, it is found in growth cones and is associated with the process of extending new axons (Fig. 2). The process is supported by other factors, such as glial cell-derived neurotrophic factors, and interventions, such as acupuncture or stem cell therapy, can accelerate regeneration and synaptophysin production [15, 49]. The nervous system and blood vessels are closely linked and often run in conjunction. Denervation of the skin can affect all phases of the wound-healing process, and decreased synaptophysin may be associated with dysfunctional wound healing [38]. In the context of DFUs and neurodegeneration, tracking synaptophysin expression can estimate peripheral nerve density and serve as a functional marker of neuronal healing efficiency.

TNFRSF10B is a receptor for the TRAIL ligand that initiates apoptosis in specific target cells, a process essential for maintaining tissue homeostasis and regulating the immune response [18]. Apoptosis clears inflammatory cells once the acute response has subsided, thereby preventing excessive tissue damage and facilitating a proper transition from inflammation to tissue repair. This controlled cell death is fundamental for resolving inflammation and enabling normal tissue remodeling and regeneration [50](Fig. 2). TNFRSF10B has complex and often negative effects on wound healing, particularly under conditions of increased inflammatory and cellular stress. It promotes apoptosis in cells, such as fibroblasts, especially in conditions like diabetes, where excessive inflammation is a determining factor (Fig. 2). Since TNFRSF10B and TNF-α are members of the TNF superfamily, it is important to distinguish their roles in nerve regeneration. TRAIL-DR5 signaling acts primarily as a deleterious mechanism that promotes neuronal apoptosis rather than regeneration in the nervous system following injury. While this pathway is involved in controlling cell turnover, its activation generally leads to neurodegeneration rather than regeneration [51]. Thus, its levels should be low for nerve regeneration. Decreased expression and inhibition of TNFRSF10B to delay damage and promote nerve regeneration is supported by our findings on diabetic wounds and other studies on spinal cord injury and traumatic brain injury [8, 51]. Given its role in apoptosis, increased TNFRSF10B expression under stress conditions may contribute to neuronal injury and impaired tissue repair. Therefore, modulating TNFRSF10B-mediated apoptotic signaling may influence wound healing by altering cell survival and tissue remodeling [17, 52]. On the other hand, TNF-α plays a complex, dual role in nerve regeneration, acting as both a critical, early mediator of repair and a potential source of neuroinflammation. While necessary for initial regeneration, immediate inhibition of excessive TNF-α can improve axonal regrowth in peripheral injuries. It often promotes repair by aiding macrophages in debris clearance and influencing neuronal survival through TNFR2 signaling [53], as supported by studies in dorsal root ganglion and sciatic nerve injury.

Nerve growth factor (NGF) is a key neurotrophic factor (protein) crucial for the development, maintenance, and survival of sensory and sympathetic neurons in the peripheral nervous system and for certain cholinergic neurons in the central nervous system. It promotes axonal growth and the synthesis and release of neurotransmitters. NGF binds to receptors on pain-sensitive nerve endings, activating signals, increasing the sensitivity of pain pathways, and increasing transmission by increasing sensitivity to painful stimuli. In inflammatory conditions, it can contribute to the development and maintenance of chronic pain. NGF also acts on immune cells, endothelial cells, and other tissues, suggesting a role in a wider range of biological processes [54, 55]. It is consistently upregulated in cutaneous wound healing, and its inhibition has been shown to delay skin repair [6, 56]. Although the precise mechanisms remain incompletely understood, NGF promotes keratinocyte proliferation and angiogenesis, even under hyperglycemic conditions [30].

Brain-derived neurotrophic factor (BDNF) is a protein crucial for neuronal survival, growth, and plasticity, and plays a vital role in learning, memory, and mood. BNDF protects neurons against injury and stimulates neurogenesis during the healing process [57]. Increased BDNF expression in wounds further contributes to repair by enhancing angiogenesis, positioning it as a central mediator of neuroregeneration [58] (Fig. 2). BDNF plays a complex role in wound repair, generally promoting nerve and tissue regeneration, but in some cases, such as in asthmatic airway epithelial cells, it can inhibit wound healing. After injury, BDNF promotes nerve regeneration by supporting the survival, migration, and differentiation of neural precursor cells, stimulating angiogenesis, and improving synaptic plasticity. For bone fractures and other tissue repair, BDNF is also involved in cell proliferation, migration, and differentiation, while in intestinal injuries, it aids in barrier restoration and mucosal healing [59].

## Impact of hyperglycemia on wound healing mechanisms

Diabetes is characterized by chronic hyperglycemia and increased oxidative stress, which can change the expression of several factors involved in nerve repair and wound healing. These changes may contribute to the chronic, nonhealing nature of DFUs and other skin complications seen in this disease. Type 2 diabetes is strongly associated with delayed wound healing, often leading to chronic DFUs that reflect underlying problems with the normal healing process [50].

Hyperglycemia has also been shown to alter the inflammatory phase of wound healing, creating a sustained pro-inflammatory environment that impairs tissue repair. Higher blood glucose has been correlated with an increased erythrocyte sedimentation rate and C-reactive protein, indicating increased inflammation [60]. Elevated glucose levels promote excessive generation of reactive oxygen species and advanced glycation end products, which chronically activate signaling pathways, leading to persistent overexpression of pro-inflammatory mediators, such as IL-6, IL-8, and TNF-α [60, 61]. This dysregulation prolongs leukocyte infiltration and delays the resolution of inflammation, contributing to ECM damage [61]. Together, these processes lead to a chronic inflammatory environment that limits the availability of growth factors, damages endothelial cells, and causes fibroblast exhaustion [62–64]. This persistent inflammation under hyperglycemic conditions prevents wounds from progressing into the proliferative and remodeling phases, making it a key component in impaired healing in diabetic wounds.

Angiogenesis is also significantly impaired in the presence of hyperglycemia. Diabetes leads to endothelial dysfunction, which reduces endothelial cell proliferation, migration, and tube formation, all of which are essential for new blood vessel growth. These changes are linked to increased oxidative stress and to upregulation of the thrombospondin (TSP)1-CD47 pathway, which together blunt the angiogenic response [65]. Neurodegeneration often parallels these vascular deficits, as hypoxic conditions further limit neuronal survival and axonal outgrowth. This combination of inadequate perfusion and neural injury creates a microenvironment that is unsupportive of both tissue and neuronal regeneration [37]. Impaired angiogenesis in diabetes has also been observed in oral wound healing, where reduced VEGF levels and abnormal vessel formation are associated with poorer healing outcomes [66]. This diminished vascular response limits oxygen and nutrient delivery to injured tissue, contributing to delayed wound closure and impaired nerve regeneration. Given the strong association between hyperglycemia and impaired angiogenesis, VEGF has been extensively studied as a therapeutic candidate to restore vascularization in diabetic wounds [37, 67].

ECM remodeling is one of the final phases of repair, restoring tissue integrity through collagen deposition, crosslinking, and structural reinforcement. Collagen is the primary structural component of the ECM, so its dysfunction directly reflects ECM dysfunction [68]. ECM remodeling, which provides structural scaffolding, regulates cell behavior, and facilitates intercellular communication [39]. ECM remodeling is also significantly disrupted in the presence of hyperglycemia. Fibroblasts from diabetic wounds exhibit abnormal gene expression and produce a weaker, fibronectin-rich matrix that fails to support proper tissue repair. These fibroblasts also respond poorly to growth factors, such as transforming growth factor (TGF)-β, contributing to defective matrix formation and organization [63]. Hyperglycemia further disrupts keratinocyte and fibroblast migration and proliferation [64, 69], impairing the cellular dynamics required for closure. In diabetes, there is increased abnormal collagen deposition and disrupted composition, which mirrors a broader pattern of ECM disorganization in diabetic tissues [70]. These changes create a structurally unstable wound environment that impairs healing and neuroregeneration [71].

Axonal regeneration, one of the main processes involved in wound repair, is commonly impaired in people with diabetes. Studies have shown that patients with diabetes have significantly reduced axon regeneration, even in those who don’t yet show clinical signs of neuropathy [72]. This suggests that impaired regeneration may occur early in the disease and could contribute to the later development of neuropathy [62]. Over time, this lack of proper axonal repair can make wounds more likely to become chronic and difficult to heal.

## Effects of hyperglycemia on the mediators of axonal regeneration

The molecular markers previously discussed as central to normal wound healing and axonal regeneration are altered under hyperglycemic conditions [8]. Activin A has demonstrated decreased expression in the chronic diabetic environment, and abnormal signaling has been linked to uncontrolled inflammation and other complications of diabetes [8, 73]. In chronic diabetic wounds, reduced activin A signaling has been associated with impaired regulation of inflammation and tissue repair. Conversely, hyperglycemia is associated with increased activin A levels in acute or non-cutaneous contexts, including conditions such as acute myocardial infarction or prediabetes and diabetes. In some cases, the elevation may represent a compensatory response to protect against inflammation and oxidative stress associated with hyperglycemia. For example, glucose can enhance activin A release from endothelial cells, thereby attenuating pro-inflammatory markers, such as IL-8 [74]. Overall, these findings suggest that activin A signaling is dependent on disease stage, tissue type, and local wound conditions, with both increased and decreased expression observed under hyperglycemic conditions. This may link its expression to either impaired or dysregulated wound healing processes.

Synaptophysin is also reduced under hyperglycemic conditions [8, 75]. This reduction reflects impaired axonal regeneration and diminished neurovascular coordination, connecting to delayed tissue recovery because synapse density and neuronal connections are critical structural and functional markers of successful nerve regeneration. A decreased synaptophysin suggests insufficient or impaired nerve regeneration. Hyperglycemia negatively affects synaptophysin, typically causing decreased protein levels and impaired function, which is linked to impaired synaptic plasticity and cognitive deficits [8, 75]. In some cases, such as in the retina, hyperglycemia can disrupt the normal post-translational modification of synaptophysin, leading to an accumulation of incompletely processed protein and subsequent accelerated degradation [75]. Synaptophysin may represent a potential target for promoting wound healing in DFUs because some of the negative effects of high glucose on synaptophysin and synaptic plasticity can be partially reversed by treatments, such as brain-derived neurotrophic factor (BDNF), which acts through pathways like the phosphatidylinositol-3-kinase (PI3K)/Akt signaling pathway [76].

TNFRSF10B is also affected by the inflammatory environment in diabetes. Hyperglycemia increases TNFRSF10B expression in non-cutaneous contexts, particularly in conditions such as diabetic kidney disease (DKD) and potentially in the retina. This upregulation is linked to high glucose-induced cellular stress, inflammation, and apoptosis, as high glucose can stimulate pathways involving FOXO1, which may influence TNFRSF10B expression [77]. However, the effects of hyperglycemia on TNFRSF10B expression during wound healing in diabetic wounds remain unexplored. Hyperglycemia prolongs the inflammatory phase of wound healing, leading to sustained macrophage activation and excessive release of proinflammatory cytokines, such as TNF [78]. This chronic inflammatory state disrupts the normal balance between cell survival and apoptosis, leading to excessive tissue injury and delayed inflammation resolution. Increased apoptosis in diabetes leads to further tissue damage and impairs the healing response [79]. Dysregulated apoptotic signaling through TNFRSF10B destabilizes the wound microenvironment and interferes with angiogenesis and ECM remodeling. These changes prevent a proper transition into the proliferative and remodeling phases of repair, ultimately delaying healing and limiting axonal regeneration.

NGF, which is normally upregulated after cutaneous injury, shows decreased expression in hyperglycemic environments [80]. This reduction weakens neuronal survival and axonal sprouting, disrupting the regenerative signaling required for proper reinnervation. Hyperglycemia in diabetes leads to depletion of NGF in peripheral nerves, contributing to diabetic neuropathy. This reduction in NGF impairs nerve regeneration and survival, leading to the sensory deficit characteristic of neuropathy, such as pain, numbness, and tingling. High blood sugar also creates a damaging environment through oxidative stress and other metabolic changes, further interfering with NGF signaling and nerve health [81]. BDNF is similarly downregulated in diabetic tissue, resulting in diminished mechanoreceptor innervation [80, 81]. Because BDNF also supports angiogenesis and neurovascular stability, its loss contributes to both impaired neuronal regeneration and inadequate vascular support during healing. Together, reduced NGF and BDNF expression under hyperglycemia disrupts the tightly regulated healing cascade, leading to impaired reinnervation and the persistence of nonhealing wounds.

## Pathways altered with hyperglycemia

Many of the signaling pathways discussed in this section have been characterized in non-cutaneous systems, and their relevance to diabetic wound healing is inferred rather than directly established. Inflammation plays a central role in the normal wound healing process, regulating tissue regeneration through immune cell activation and signaling [82]. In diabetes, this regulation is disrupted by persistent low-grade inflammation, marked by elevated circulating cytokines such as TNF-α, IL-6, and C-reactive protein (CRP), which are associated with poor glycemic control and increased complications [83]. This chronic inflammatory state alters the wound microenvironment early in the healing cascade, affecting downstream pathways involved in angiogenesis, ECM remodeling, and axonal regeneration [37, 39, 75, 80]. These inflammatory changes are closely intertwined with molecular signaling networks that regulate these processes, including pathways mediated by Activin A and TNFRSF10B [10, 18]. Disruption of these mediators represents a key mechanism underlying impaired healing with hyperglycemia [78].

Follistatin is a key inhibitor of activin A signaling, binding activin A and preventing its interaction with the activin receptor complex [84]. Under hyperglycemic conditions, circulating levels of follistatin are significantly elevated compared with those in normal controls [85]. This increase in follistatin suppresses activin A activity, blunting downstream signaling that normally promotes angiogenesis, ECM remodeling, and inflammatory resolution. As a result, activin A’s ability to support vascularization, stabilize the ECM, and facilitate neurovascular repair is impaired, contributing to delayed healing and chronic wound formation. Studies show that reduced decapentaplegic homolog 2 (SMAD2) activation can be associated with more favorable metabolic outcomes in diabetes. For example, decreased SMAD2 signaling has been linked to improved glucose tolerance and insulin secretion in experimental animal models [86, 87]. Conversely, increased SMAD2 activity has been observed in tissues affected by diabetic complications such as nephropathy [88], indicating a potential role in pathological remodeling. This suggests that while SMAD2 signaling downstream of activin A is beneficial for tissue repair, dysregulated activation in diabetes may contribute to disease progression. Elevated follistatin levels in hyperglycemia likely dampen activin A activity and reduce SMAD2 signaling. A decreased activin A activity will impair wound healing, as suggested by our results of decreased activin A in diabetic wounds [8].

Synaptophysin serves as a marker of neuronal density [13], and its decreased expression under hyperglycemia reflects impaired neuronal regeneration rather than driving it, indicating reduced synaptic integrity and axonal remodeling. Mediators of synaptophysin signaling include dynamin and SNARE proteins such as synaptobrevin, which work with synaptophysin to regulate neurotransmitter release. Synaptophysin’s interaction with dynamin, for instance, appears to control the amount of neurotransmitter released per vesicle, while its interaction with SNARE proteins is crucial for the exocytosis process itself [89]. Dynamin is crucial for cellular processes like cell migration and wound closure by regulating the actin cytoskeleton. Hyperglycemia impairs dynamin function, which is vital for pancreatic beta cell function and glucose homeostasis. In hyperglycemia, dynamin 2 is less efficient at coupling exocytosis and endocytosis, leading to impaired insulin secretion and glucose intolerance. Additionally, some dynamin-related proteins exhibit altered expression, such as a downregulation of dynamin-like protein 1 in certain tissues, contributing to the negative effects of hyperglycemia [90, 91]. This suggests that altered dynamin may affect wound healing by altering axonal regeneration. This notion is supported by the findings of Vijayaraghavan et al. that altered dynamin (particularly DYN-1 in *C. elegans* and dynamin 2/DNM2 in mammals) affects wound healing by impairing axonal regeneration and repair mechanisms. Studies show that dynamin acts as a key mediator in regenerative axonal fusion, a process that reconnects severed nerves and controls the availability of fusogen proteins (like EFF-1) required to reestablish neuronal continuity post-injury [92]. It is also important to note that dynamin is involved in the recognition of apoptotic cells, a process that is repurposed to help recognize and repair broken axon fragments [93]. It is important to note that dynamin is involved in mitochondrial damage, defective mitophagy, and NLRP3 inflammasome activation (mice study) [94] and inflammatory responses in human periodontal macrophages via interaction with hexokinase 1 [95]. These findings indicate the role of dynamin in regulating inflammation. Moreover, a significant increase in Dynamin-related protein 1 (Drp1) expression drives excessive mitochondrial fragmentation, caspase-3 activation, and ROS generation with hyperglycemia [96]. The implications of these findings should be investigated in the context of wound healing in diabetes.

SNARE proteins are crucial for wound healing because they mediate membrane repair, which is essential for cell survival after a membrane is damaged. They function by forming a complex that promotes the fusion of vesicles with the damaged plasma membrane, allowing for the repair of the cellular breach. In hyperglycemia, SNARE protein expression can be dysregulated, with some studies showing decreased levels of syntaxin-1 A and SNAP-25 in pancreatic beta-cells, which impairs insulin secretion. Other studies indicate an increase in different SNAREs, such as SNAP23, in skeletal muscle associated with insulin resistance and reduced insulin sensitivity. The effects can vary depending on the specific SNARE protein, the tissue type, and whether the cause is chronic hyperglycemia itself or the associated hyperinsulinemia [97, 98]. The relevance of SNARE protein in wound healing is supported by the fact that SNARE proteins, specifically the RIC-4/SEC-22/SYX-2 complex, in *C. elegans*, are essential for membrane fusion during cellular repair, actively promoting wound healing by fixing damage in the plasma membrane. They function by recruiting proteins to the wound site and forming a complex that seals membrane breaches, which is crucial for survival in damaged tissues and epidermal wound healing [97]. Much of this evidence is derived from non-cutaneous systems, including neuronal and metabolic tissues. These findings highlight the importance of vesicle trafficking pathways in cellular function and repair. Alterations in dynamin and SNARE protein expression may disrupt synaptic vesicle dynamics, with changes in synaptophysin reflecting underlying neuronal dysfunction rather than directly mediating wound healing. The implications of these findings in DFUs warrant further research.

Activated TNFRSF10B recruits adaptor proteins and initiates caspase signaling, regulating cell death and maintaining homeostasis [16]. The TRAIL ligand is protective against diabetes, and its deficiency is associated with reduced insulin levels [99]. These effects occur under normal or regulated TRAIL signaling. In contrast, excessive DR5 expression, as seen in hyperglycemia with uncontrolled inflammation, most likely leads to dysregulated apoptosis and increased tissue injury. Fas-associated death domain protein (FADD) acts as a key adaptor in DR5 signaling and regulates metabolism [100]. Phosphorylation of FADD suppresses insulin-degrading enzyme expression, contributing to insulin control [101]. Dysregulation of the TRAIL–DR5–FADD pathway under hyperglycemia ultimately promotes excessive apoptosis and worsens metabolic dysfunction, which can indirectly impair wound healing. FADD is a crucial regulator of programmed cell death (apoptosis and necroptosis) in wound macrophages and keratinocytes, ensuring proper healing. Combined deficiency of FADD and RIPK3 in macrophages severely delays wound healing by preventing the timely clearance of Ly6C^high^ macrophages from damaged tissue. FADD, alongside Caspase-8 and RIPK3, mediates cell death that clears pro-inflammatory macrophages from wounds, allowing for effective repair. Disruption of these pathways causes excessive accumulation of inflammatory cells in the wound, leading to impaired remodeling and impaired wound closure. FADD acts as a critical inhibitor of necroptosis (programmed necrosis) in epidermal keratinocytes. Its absence causes severe skin inflammation and defects. Further, FADD protects against TNF-driven skin lesions, ensuring that wound repair is not disrupted by chronic inflammation [102]. These findings suggest the role of FADD in wound healing. Hyperglycemia acts as a stressor that typically induces the expression and phosphorylation of FADD protein, triggering apoptosis and inflammatory pathways, particularly via the FADD-dependent, caspase-8 mediated pathway. This mediates ER stress and proapoptotic kinase activation [101]. This suggests that increased FADD in hyperglycemic conditions increases stress, which may be detrimental to wound healing, but this implication warrants investigations.

## Network analysis revealed an interacting relation

**Fig. 3:**
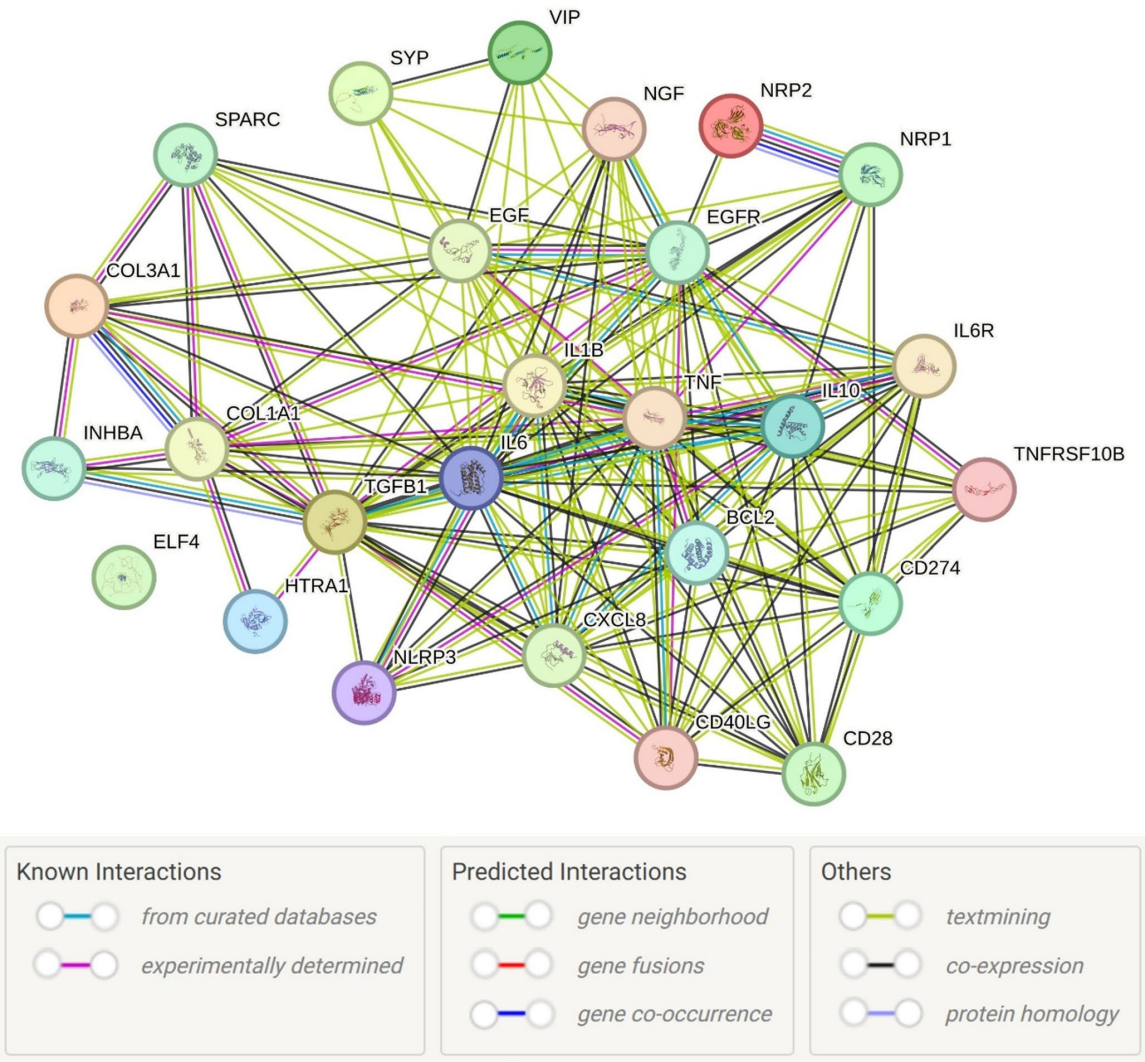
Protein-protein interaction (STRING network) predicting interaction between various mediators associated with angiogenesis, ECM remodeling, inflammation, and axonal regeneration

We used the various proteins involved in wound healing mechanisms discussed above as an input and did the network analysis. Search Tool for the Retrieval of Interacting Genes/Proteins (STRING) database of known and predicted protein-protein interactions, including direct (physical) and indirect (functional) associations stemming from computational prediction, showed interaction between various mediators associated with angiogenesis, ECM remodeling, inflammation, and axonal regeneration (Fig. 3). Network analysis showed protein-protein interaction between mediators of inflammation (IL6, IL1B, TNF, CXCL8, CD40L, NLRP3, IL10), growth factors (PDGF, FGF, EGF, EGFR, NGF), activin A (INHBA), synaptophysin (SYP), ECM components (COL1A1 and COL3A1), angiogenesis (VIP increasing VEGF expression), NRP1 (Neuropilin-1) and NRP2 (Neuropilin-2). NRP1 and NRP2 are closely related cell surface receptors crucial for guiding nerve growth and angiogenesis by acting as co-receptors for Semaphorins and VEGF. These interactions suggest that these mediators, which are involved in wound healing and axonal regeneration, interact (or may interact) with each other (Fig. 3). It should be noted that these interactions do not directly prove biological interaction or association between these proteins with wound healing or DFU healing, but support potential functional connectivity within DFU wound beds. The role of these proteins must be investigated in the context of DFU healing. This interaction strengthens the hypothesis that axonal regeneration plays a critical role in wound healing, and hyperglycemia has an effect on its expression, affecting wound healing, as discussed in this review.

It should be noted that STRING analysis shown in Fig. 3 does not provide direct evidence of interaction of the factors of interest in the context of wound healing, or how hyperglycemia may disrupt these interactions. But these findings are of interest as we have discussed the effects of hyperglycemia on most of these mediators in this review. For example, there is an interaction between proinflammatory cytokines (IL-6, IL-1β, TNF, CXCL8 (IL-8), and NLRP3 with collagens and other factors involved in wound healing, and it is well known that diabetes induces a pro-inflammatory environment. Thus, these findings, in part, support the notion that hyperglycemia may impair wound healing involving nerve regeneration.

## Limitations and knowledge gaps

Despite growing interest in the role of axonal regeneration in wound healing, several important knowledge gaps remain. Much of the evidence discussed is derived from non-cutaneous tissues and related disease models, which may not fully reflect the biology of diabetic wound healing. In addition, the relationship between impaired axonal regeneration and nonhealing DFUs remains largely associative, and direct causal evidence is still limited. There is also a lack of longitudinal human DFU data and insufficient cell-specific profiling within the wound bed, making it difficult to define how these pathways change over time and across different cellular compartments. Future studies should focus on DFU-specific models and translational approaches to determine whether targeting neuroregenerative pathways, alone or in combination with angiogenic or anti-inflammatory therapies, can improve healing outcomes. Another area of research may be molecular mechanisms of failure (which mediators(s) are significantly involved), the role of epigenetic changes, and the reason for the inability of nerves to reinnervate target tissues properly. Poor target reinnervation may be due to dysfunctional nerve regeneration. The precise mechanism behind this “abortive” sprouting (initially regenerates but does not innervate), which inhibits functional repair, is not fully elucidated. Further, specific mechanisms through which cutaneous sensory nerves communicate with keratinocytes and fibroblasts (cell-cell interaction) to drive repair are poorly understood, particularly in humans. Finally, the effects of hyperglycemia and chronic inflammation on these aspects warrant future research.

## Conclusion

Hyperglycemia and oxidative stress disrupt the molecular mediators essential for axonal regeneration, impairing inflammation, angiogenesis, and ECM remodeling. The resulting failure of nerve repair contributes to neuropathy and recurrent trauma, establishing a cycle of chronic nonhealing in DFUs. Recognizing impaired axonal regeneration as a significant component of DFU pathology emphasizes the need for therapies that can restore the normal healing process and promote neuroregeneration. Future directions should prioritize interventions that enhance neurotrophin signaling, stabilize ECM remodeling, and support angiogenesis to break the cycle of recurrent DFUs to improve long-term healing outcomes.

## Data Availability

No datasets were generated or analysed during the current study.
